# Environmental surveillance of bacteria in a new intensive care unit using plate sweeps

**DOI:** 10.1099/mgen.0.001597

**Published:** 2026-01-13

**Authors:** Aasha McMurray-Jones, Kirsten Spann, Prasad K. D. V. Yarlagadda, Jeremy Fernando, Leah W. Roberts

**Affiliations:** 1 Centre for Immunology and Infection Control, School of Biomedical Sciences, Queensland University of Technology, Brisbane, Australia; 2 School of Engineering, University of Southern Queensland, Springfield, Queensland 4350, Australia; 3 St Vincents Private Hospital, Toowoomba, Australia; 4 Rural Clinical School, University of Queensland, The Medical School, Toowoomba, Australia; 5 The University of Queensland, UQ Centre for Clinical Research, Herston, QLD 4029, Australia; 6 Australian Infectious Disease Research Centre, University of Queensland, Brisbane, Australia

**Keywords:** intensive care unit, metagenomics, plate sweeps

## Abstract

The hospital environment plays a critical role in the transmission of infectious diseases. Surveillance methods often rely on selective enrichment or deep metagenomic sequencing, which both have significant drawbacks in terms of community resolution and cost. Plate sweeps provide a practical moderate approach to cultivate a wide range of bacteria, capturing more diversity than a single colony pick without high sequencing costs. Here, we use this approach to characterize a newly built hospital intensive care unit (ICU) in Queensland, Australia. Between November 2023 and February 2024, we sampled 78 sites within an 8-bed private hospital ICU pre- and post-patient introduction to the environment. Samples were enriched on non-selective media before DNA was extracted from whole plate sweeps and sequenced using Illumina. We assessed species, antimicrobial resistance (AMR) genes, virulence genes and transmission across all samples and between the pre- and post-patient samples using Kraken2, AbritAMR and Tracs. While the rate of positive microbial growth within the ICU environment did not change significantly pre- and post-patient introduction, the post-patient microbiome consisted of largely different bacterial species; of 22 genera identified, only 3 genera were represented at both timepoints. Post-patient samples were enriched in AMR genes, including resistance to fosfomycin, quinolones and beta-lactams. Common genera identified post-patient were *Pseudomonas*, *Delftia* and *Stenotrophomonas*, often associated with areas of plumbing. Cluster analysis identified 17 possible transmission links from a single timepoint, highlighting several areas in the ICU (e.g. communal bathrooms) as key areas for transmission. We demonstrate the utility of plate sweeps as a means of economical non-selective environmental surveillance and highlight their ability to identify hotspots of transmission within a hospital ward that could be targeted by infection control prior to an outbreak of a more serious pathogen.

**Impact Statement**

Hospital infections are a growing problem, with cases in Australia nearly doubling in recent years. High-touch surfaces and plumbing can spread harmful bacteria, and antibiotic resistance makes infections harder to treat and more expensive. Our study tested a new way to monitor hospital environments by sampling surfaces in an intensive care unit (ICU) before and after patients arrived. Instead of only checking for specific resistant bugs (as hospitals usually do), we used a broader approach that captures all bacteria on a plate and then sequences their DNA. This revealed that after patients arrived, the ICU microbiome completely changed and became more resistant to antibiotics. We also tested a new tool called Tracs to track how bacteria spread between surfaces. This helped identify ‘hot spots’ that need extra cleaning to prevent outbreaks. Overall, a simple change in testing could give hospitals better insight into hidden risks, helping stop dangerous infections before they spread.

## Data Summary

Reads (post-human read removal) have been uploaded to the SRA under Bioproject PRJNA1310799. Accessions are provided in Dataset S1. Methods, including tools, version numbers and parameters, have been included in the relevant section.

## Introduction

Healthcare-associated infections (HAIs) are a continuing burden in hospital settings. Annually between 2010 and 2016, ~83,000 patients were diagnosed with HAIs in Australia [1], increasing to ~170,000 in 2018 [2], demonstrating the growing impact of HAIs on Australian patients. Compared to other complications resulting from hospitalization, infections represent the greatest burden, accounting for 38% of hospital-acquired complications in 2023–2024 [3].

The environment is a key element in seeding and progressing infectious outbreaks in healthcare settings, particularly high-touch (e.g. door handles, keyboards) and plumbing-related areas (e.g. bathrooms, sinks) [4,5]. The rise of antimicrobial resistance (AMR) only exacerbates the threat of HAIs, straining our health resources as a consequence of prolonged inpatient stays [6], intensive infection control requirements and extra treatment costs [7,8].

**Fig. 1.**
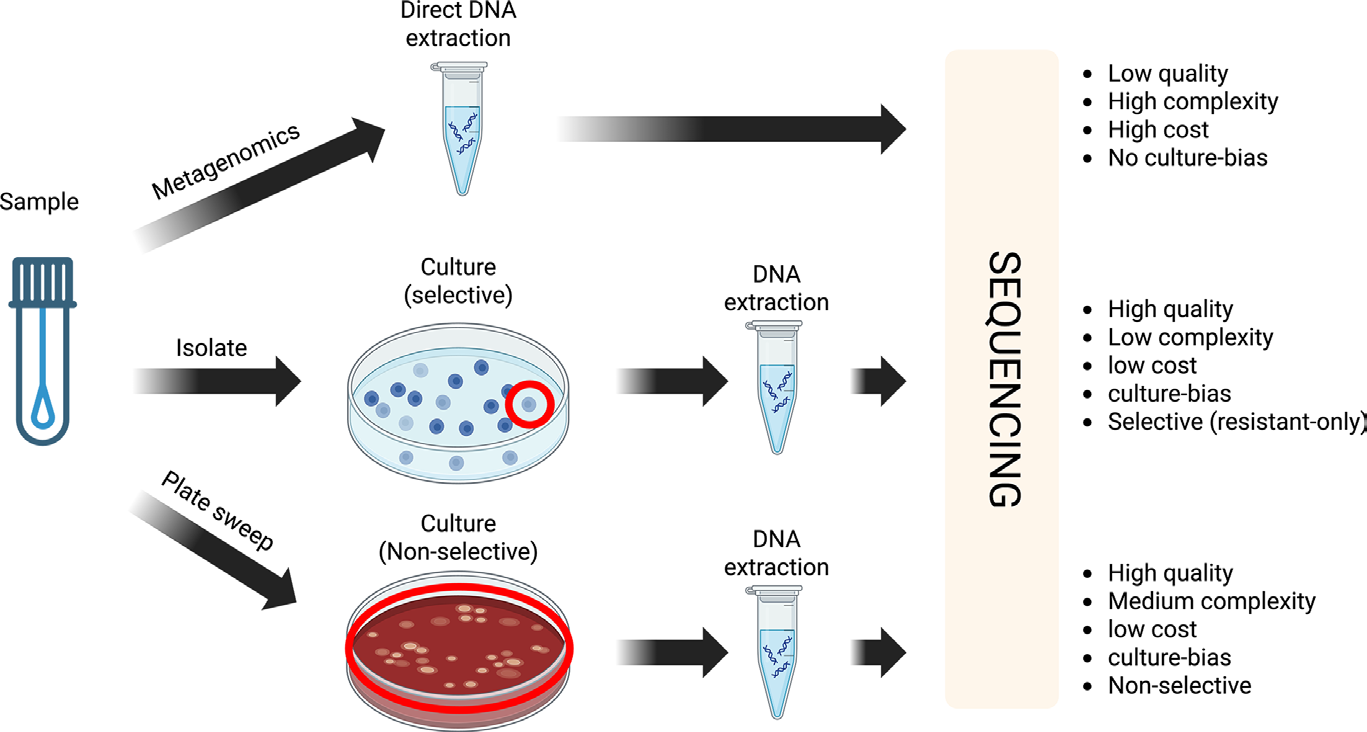
Advantages and disadvantages of environmental surveillance techniques in hospital settings.

Current environmental surveillance in hospital settings is largely performed phenotypically from single colony picks on media enriched for AMR (Fig. 1). This has several limitations, including (i) culture-bias, where only organisms cultivatable in a laboratory setting are identifiable, (ii) resistance-bias, which limits our ability to understand resistance emergence over time and (iii) limited resolution to track the larger diversity of microbes present at a sampling site. More recently, healthcare settings have utilized metagenomic sequencing directly from environmental samples to capture a complete picture of the microbial community [9]. However, adoption of this method for routine surveillance has been slow, due to prohibitive costs, the complexity of analysing the data and low-biomass sampling from the hospital environment [9].

As an alternative to these methods, plate sweeps (also referred to as ‘quasi-metagenomics’) have emerged as an accessible and cost-effective intermediate for understanding bacterial diversity from an environmental sample [10,11]. While plate sweeps are unable to provide a comprehensive community profile (i.e. they still retain culture-bias), most pathogens of clinical interest in the environment are readily culturable [12,13]. Non-selective growth conditions can enrich for a range of bacteria, and DNA extracted from the plate requires less sequencing depth compared to metagenomics. While similar studies have been conducted in patient cohorts to detect transmission of key pathogens using culture-based deep sequencing [14], to our knowledge, only one other study has utilized plate sweep techniques for whole genome sequencing (WGS) to characterize the hospital microbiome through untargeted surveillance [15]. This approach could provide an important practical step in routine hospital surveillance. Here, we conducted a pilot study of the hospital environment using plate sweeps to understand the diversity of environmental bacteria pre- and post-patient introduction.

## Methods

### Hospital setting

St Vincent’s Private Hospital, Toowoomba, is a regional private hospital with ~200 hospital beds. The newly developed intensive care has eight beds, including two negative pressure rooms. The main case mix of the ICU includes respiratory failure, sepsis, post-operative care for orthopaedic, ear, nose and throat and general surgery. The unit admits ~500 patients per year.

### Sampling and storage

Seventy-eight sites were sampled within the new ICU and collected once before patient use (23.11.23 – timepoint 1; T1) and once after patient/staff introduction (22.01.24 – timepoint 2; T2) using standard cotton sampling swabs moistened with sterile water (total samples for T1 and T2=156). This included samples from high-touch areas (doorknobs, bed frames, sinks, toilets and medical equipment), areas expected to have high biomass (plumbing and drains) and areas that are infrequently/hard to clean, including keyboards used by staff. Samples were stored in 1 ml of Luria–Bertani (LB) broth at 4 °C for up to 48 h.

### Culturing, DNA extraction and DNA sequencing

Briefly, 50 µl of swab-LB broth suspension was aliquoted onto each of three agar plates [nutrient (NA), MacConkey (MAC) and Horse Blood agars (HBA)] and incubated at 37 °C overnight. Next, 2 ml of LB broth was added to plates, and cultures were resuspended using plate spreaders. Briefly, 500 µl of resuspended plate sweep was used to extract DNA using DNeasy PowerSoil Pro Kit (QIAGEN) as per manufacturer’s instructions. DNA was sequenced using an Illumina NovaSeq 6000 at the Centre for Microbiome Research, QUT.

### Bioinformatic analysis

Raw sequencing reads were checked for quality using FastQC [16] (v0.12.1) and trimmed using fastp [17] (v0.23.4) with ‘--average_qual 20 --length_required 80’ parameters. Human reads were removed using nohuman [18] (v0.1.1) at default settings. Seqkit [19] (v2.8.2) was used to assess read metrics pre- and post-filtering.

Trimmed reads were *de novo* assembled using MetaSPAdes [20] (v3.15.5) and binned using Metabat2 [21] (v2 : 2.15), at default settings. Species were identified from trimmed reads and binned metagenome-assembled genomes (MAGs) using Kraken2 [22] (v2.1.3) and the Kraken2 standard database (20240112). Relative proportions were calculated based on the number of observed samples containing X divided by the number of all sampled sites. Completeness and contamination for each binned MAG were estimated using CheckM2 [23] (v1.0.1). AMR and virulence genes were identified using AbritAMR [24] (v1.0.17). Genomad ‘end-to-end’ (v1.11.2) [25] at default settings with database v1.9 was used to classify MAGs contigs as chromosome, virus or plasmid. Clusters were identified using Skani [26] (v0.2.1) at a pairwise average nucleotide identity (ANI) threshold≥99.95 for binned MAGs.

Tracs [27] (v1.0.1) was used as an end-to-end pipeline for estimating transmission from metagenomic samples. Trimmed and human decontaminated reads from timepoint 2 only were aligned to the Genome Taxonomy Database (GTDB) (gtdb-rs214-reps.k51.sbt.zip) using the ‘--keep-all’ flag. Two samples (ICU_S39 and ICU_S49) failed as there were zero kmers which aligned to GTDB [28]. Aligned samples were then combined into multiple sequence alignments according to their respective reference genome, and distance estimation was performed using the parameters ‘--snp_threshold 1000 --filter’. Clusters were then inferred with the parameters ‘--distance filter --threshold 10’.

## Results

### Plumbing remains a primary source of bacteria in the hospital environment

Seventy-eight sites around the ICU were sampled, once for timepoint 1 (pre-patient introduction T1) and again for timepoint 2 (post-patient introduction T2). Positivity was measured by observing bacterial growth on any media after overnight culture at 37 °C. Several media choices were selected to determine the most appropriate non-selective media for routine surveillance. The amount of growth on NA and HBA was comparable at each site and timepoint, with higher species diversity on the HBA plates (Dataset S1).

Overall, 24/78 sites (31%) were positive in T1, and 44/78 sites (56%) were positive in T2. Sample locations were broadly split into the following three categories:

1. Patient room (including beds, room handles, room equipment and room switches),
2. High-touch communal surfaces (including all areas in the reception, the laundry trolley and the medicine room handle) and
3. Plumbing-related (all sinks, toilets, showers and drains in both communal and private patient rooms, including door handles to those rooms).

**Fig. 2.**
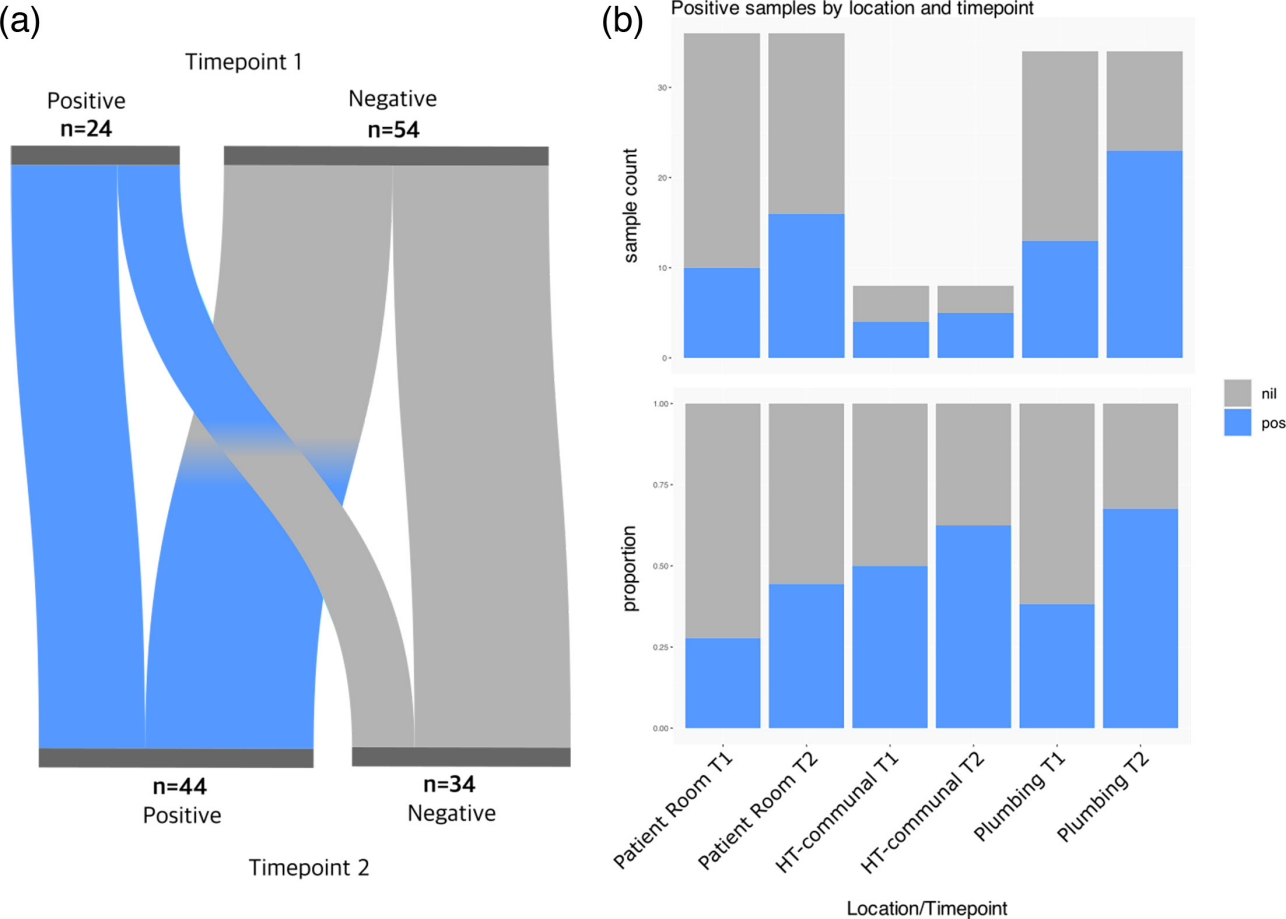
Positive samples by location across timepoints 1 and 2. (**a**) A Sankey plot showing change in site positivity/negativity or no change from timepoint 1 to timepoint 2. (**b**) Sample counts that were positive or negative for bacterial growth at timepoint 1 (**T1**) or timepoint 2 (**T2**) by general location [patient room, high-touch (HT) or plumbing]. Nil=no growth, pos=positive growth.

**Fig. 3.**
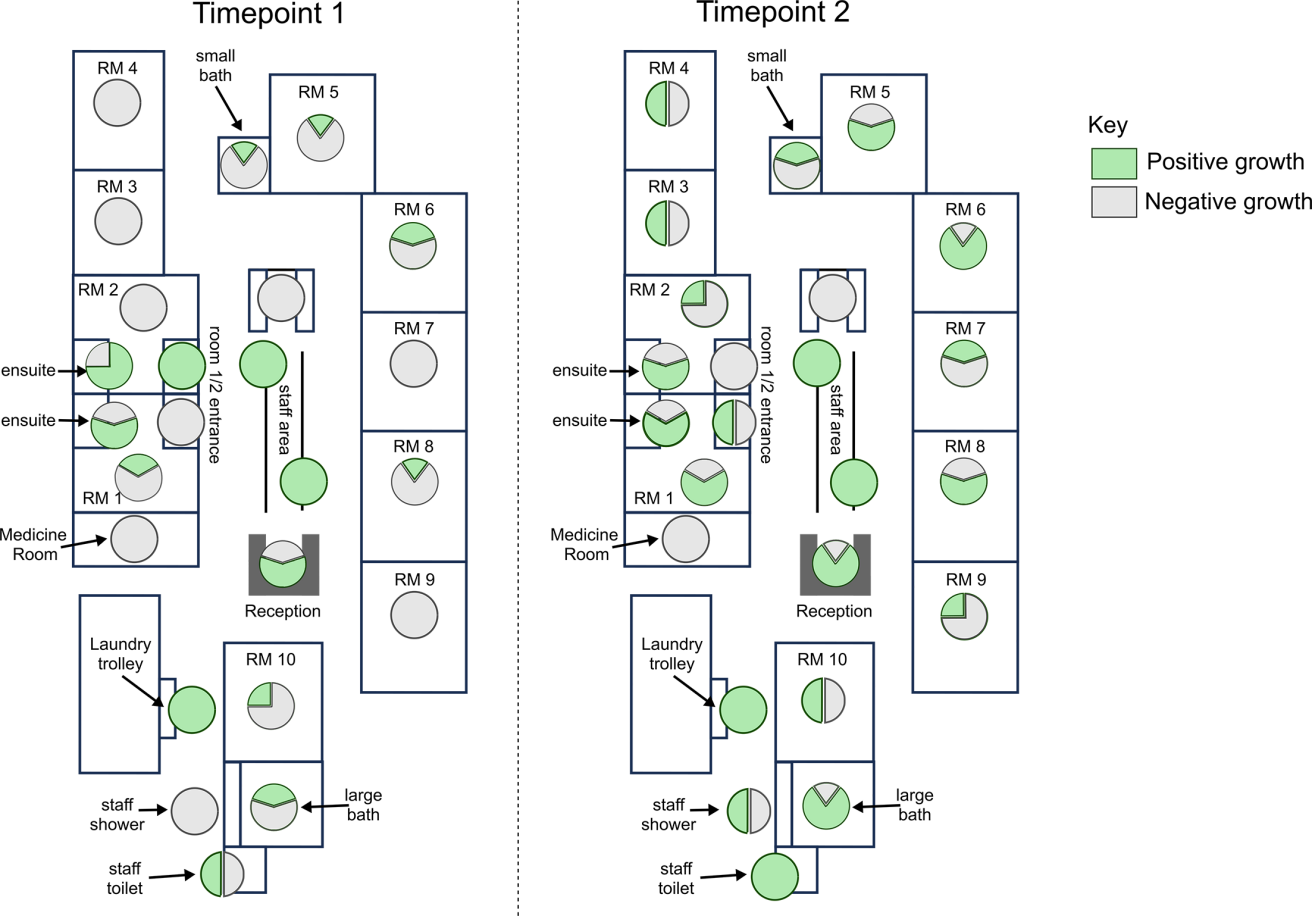
Site positivity between T1 and T2: pie charts showing the proportion of samples from specific regions of the ICU with positive (green) or negative (grey) bacterial growth. Timepoint 1: pre-patient introduction. Timepoint 2: post-patient introduction. RM=patient room. RM 1 and 2=negative pressure rooms.

Across all three categories, there was an increase in site positivity from T1 to T2 (Fig. 2). Plumbing-related sites had both the highest positivity rate at both timepoints and the highest increase in positive sites from T1 to T2 (Fig. 2). Across T1 and T2, 25 sample sites remained negative at both timepoints and were mainly door handles and switches (18/25, 72%; Fig. 3). Importantly, 15 positive sites remained positive and included bathroom facilities (shared 4/15; or in private rooms 3/15) and high-touch communal areas (4/15). Importantly, 29 negative sites (mainly plumbing-related, 16/29; or patient room-related, 12/29) became positive, while 9 positive sites (mainly handles and rails in patient rooms and communal areas, 5/9) became negative (Fig. 3, Dataset S1).

### Sequencing revealed abundant changes in the environmental microbiome after patient introduction

Positive samples were selected for sequencing based on the abundance and type of growth on the media. Of the 24 positive sites for bacterial growth in T1, 16 were sequenced (due to project constraints; see Discussion), and of the 44 positive sites for T2, 41 were sequenced (due to insufficient DNA; Dataset S1). To test the choice of media, bacteria from all three media types for two samples were sequenced to compare diversity. There was no significant difference in genera identified amongst media types when typing with the Kraken2 standard database (Table S1, available in the online Supplementary Material). As such, NA media was preferred for sequencing except where HBA or MAC had higher visual diversity or growth (Table S1). In addition, filtering to remove human reads had a negligible effect on the total read counts (Dataset S1).

For T1, 10/16 sample sites could be typed to either the genus or species level, filtering for genus/species assignments above 5%. The exceptions were ICU_S01, ICU_S03, ICU_S05, ICU_S06, ICU_S08 and ICU_S10, which were unable to be classified (>90% unclassified).

**Fig. 4.**
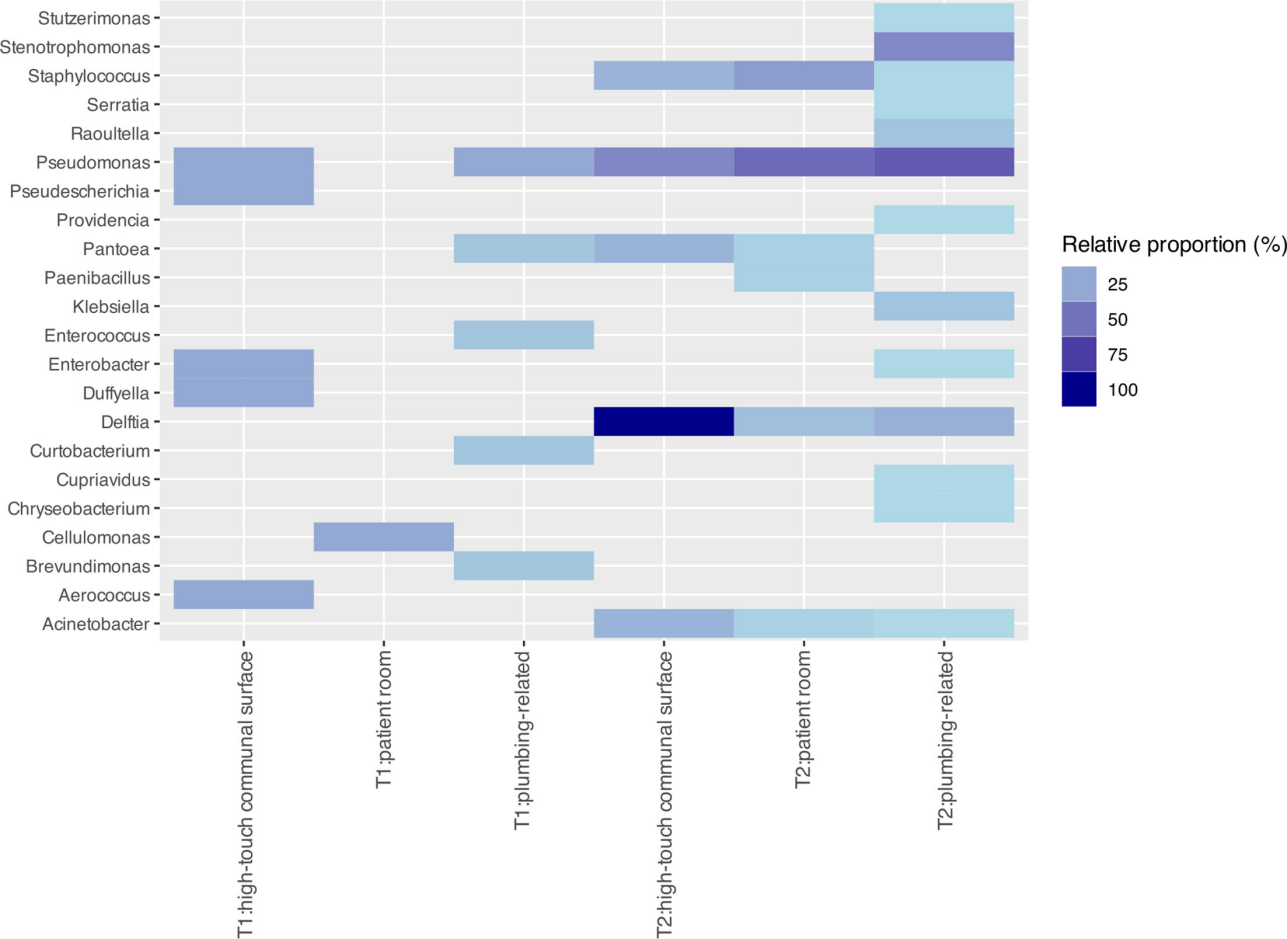
Genus identified per location: relative proportion (%) of genera identified across the three broad sampling site categories in timepoint 1 (**T1**) and timepoint 2 (**T2**).

Genera identified from T1 were mainly associated with environmental bacteria that have not been routinely reported to cause pathogenesis in humans. This includes *Duffyella*, *Brevundimonas* and *Cellulomonas* (Fig. 4). However, some genera associated with opportunistic infections were also detected, including *Aerococcus*, *Enterococcus* and *Pseudomonas*.

At T2, 2 months after patient and staff introduction, we observed a large shift in the microbial environment of the ICU (Figs4  5). Not only were the bacterial residents different, but the rate of positive growth in all areas of the ICU was higher than before patient introduction (Fig. 2). Of 41 sites, 39 were positive for at least one bacterial genus at >5% abundance. Two sites (ICU_S39 and ICU_S49) were unable to be classified.

**Fig. 5.**
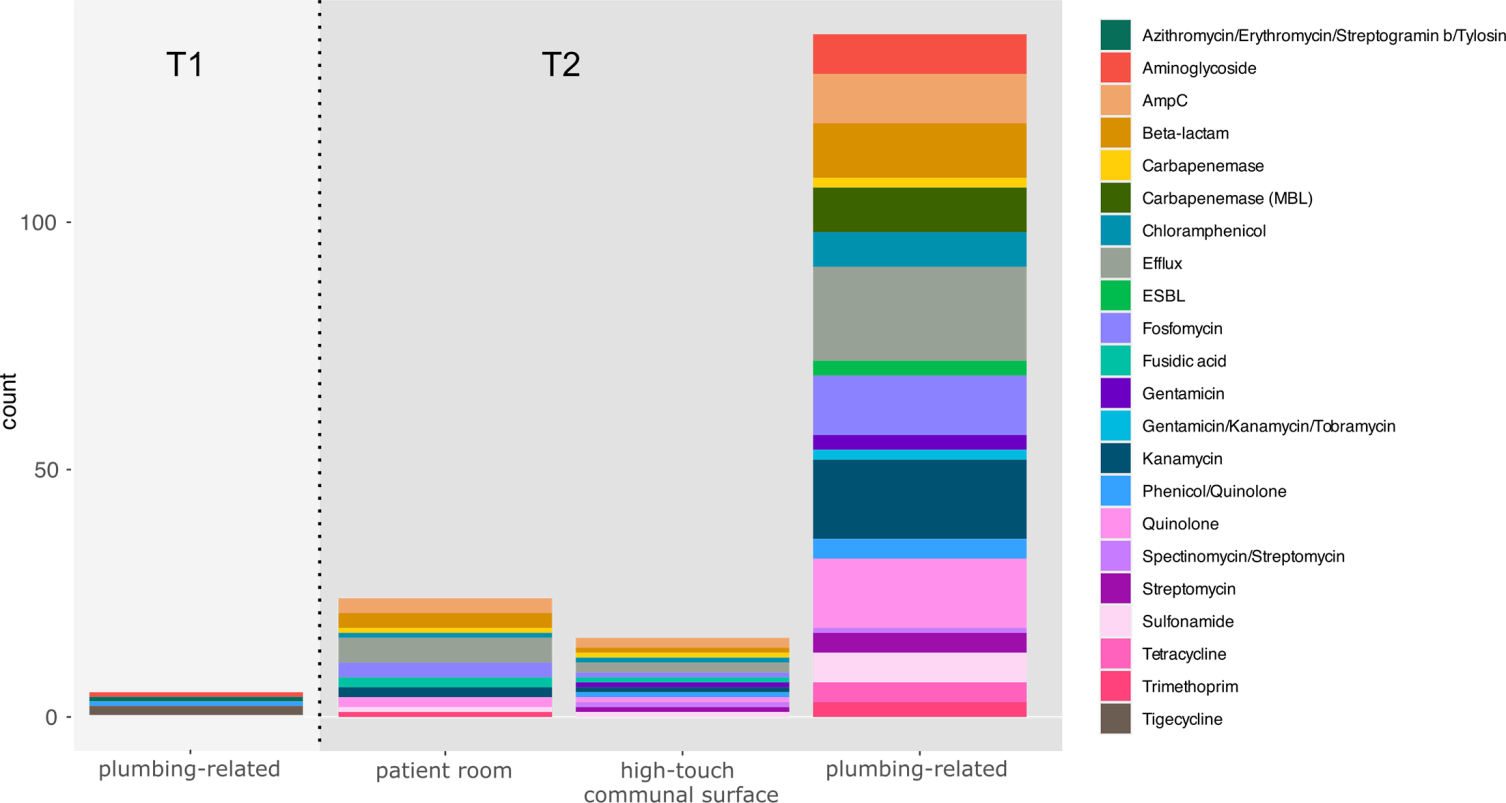
T1 vs. T2 AMR: number of identified genes across a range of AMR categories detected in T1 and T2 from the three broad sampling site categories.

Twelve additional genera were identified at T2 compared to T1, including well-known human pathogens such as *Acinetobacter*, *Klebsiella* and *Staphylococcus*. We identified an abundance of *Pseudomonas* (23/41, 56%) and *Stenotrophomonas* (9/41, 22%), which can harbour resistance to last-line antibiotics such as carbapenems and are frequent residents of hospital environments. Only three genera, *Pseudomonas*, *Pantoea* and *Enterobacter*, were represented at both timepoints (Fig. 4). Based on the overall number of sites sampled and sequenced at each timepoint, the relative rate of *Pantoea* and *Enterobacter* at T2 was determined to be 78 and 39% of T1, respectively. Conversely, *Pseudomonas* was estimated to be threefold higher at T2 compared to T1.

Of four locations where we had paired T1 and T2 sequencing with positive growth (keyboard 2, laundry trolley, room 1 toilet and room 2 bathroom sink), none had evidence of establishment of the initial T1 microbial community, demonstrating complete displacement by T2. *Pseudomonas* was the dominant genus across all locations, followed by *Stenotrophomonas* and *Delftia* (Fig. 4).

Few AMR and virulence genes were identified in T1 samples and only from plumbing-related sites (*n*=4, 25%; Figs 5 and S1). Two samples [ICU_S07 (room 2 bathroom sink) and ICU_S14 (communal bathroom sink)] carried the tigecycline-resistance gene *tmexD2*, a resistance-nodulation-division family efflux pump. Only two additional samples carried resistance genes; ICU_S15 (room 2 sink) carried *aac(6′)-I* and *msr(C)*, conferring resistance to aminoglycoside and azithromycin, and ICU_S16 (room 1 toilet) carried *oqxB9*, conferring quinolone resistance. Interestingly, we detected a high prevalence of virulence genes, which consisted mostly of resistance to metals (56% of samples). This included copper (*copB*), mercury (*merABDEFPRT*), silver (*silACPR*), tellurite (*terD*) and divalent metal (*fieF*) resistance. Two genes associated with resistance to cleaning agents, *qacH* and *qacE*, were identified in two samples [ICU_S01 (room 6 bed) and ICU_S07 (room 2 bathroom sink)]. Overall, at T1, 75% of samples (*n*=12) had no resistance genes detected. Briefly, 44% (*n*=7) had no virulence genes nor AMR genes.

Binning of *de novo* assemblies for each sample using Metabat2 enabled closer approximation of the species carrying AMR and virulence genes. Binning of T1 samples identified 111 bins, with 22% (*n*=24) above 50% completion. *tmexD2* was identified in two *Pseudomonas* sp., while the *aac(6′)-I* and *msr(C*) genes were identified in an *Enterococcus faecium*, and the *oqxB9* gene was identified in *Pantoea agglomerans*.

In contrast to T1, T2 revealed the introduction of substantially more AMR and virulence genes (*n*=38, 93%; Figs 5 and S1). Efflux pumps were identified in >60% samples, followed by resistance to kanamycin (46%), quinolones (41%), fosfomycin (39%) and beta-lactams (36%). Most samples (80%) carried resistance to metals, followed by biocide resistance (65%). Genes involved in pathogenicity (iron acquisition, biofilm formation) remained rare (*n*=3). Ten samples (24%) had no AMR genes identified. Five samples had no virulence genes identified, and three samples (ICU_S39 reception bench, ICU_S49 room 5 bed and ICU_S56 room 6 light switch) had neither AMR nor virulence genes.

**Fig. 6.**
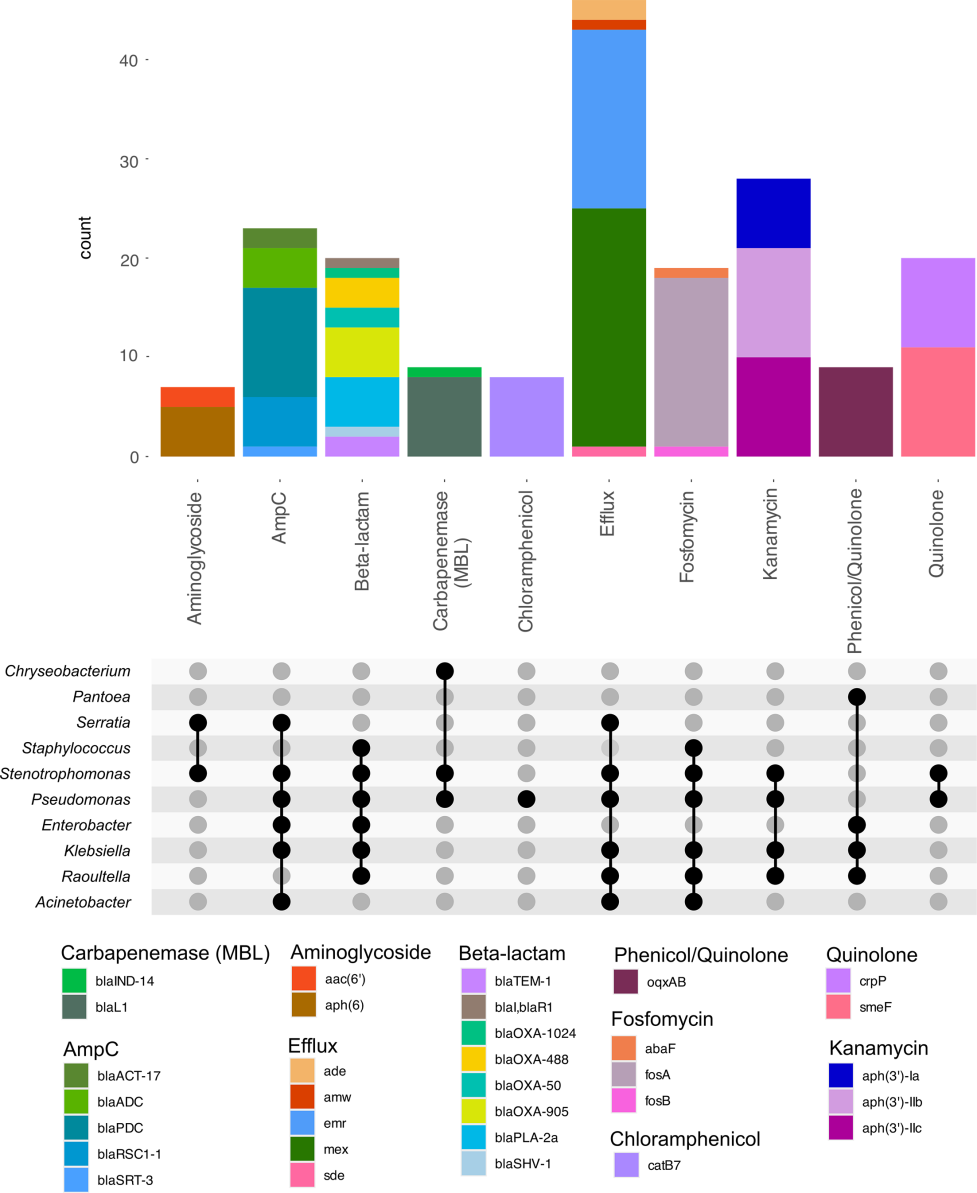
T2 AMR detailed binning: detailed analysis of AMR genes and predicted associations with genera.

Once again, binning allowed closer approximation of the species contributing AMR and virulence genes to the sample (Fig. 6). In T2, we identified 296 bins, with 43% (*n*=128) above 50% completion. The efflux pump *mexAEX* was only identified in bins primarily consisting of *Pseudomonas* sp., while *emrABC* was identified in *Stenotrophomonas maltophilia* bins. *S. maltophilia* also appeared to be associated with the carriage of *aph(3′)-IIb* and *smeF*, conferring kanamycin and quinolone resistance. Conversely, *fosA*, *catB7* and *crpP* carriage were more associated with *Pseudomonas aeruginosa*, although some *Klebsiella* species (*Klebsiella pneumoniae* and *Klebsiella michiganensis*) and *Raoultella planticola* also appeared to carry *fosA* along with the efflux *oqxAB*.

In terms of virulence, *Delftia* sp. was most often associated with mercury resistance (*merADEPRT*), while copper resistance (*copABLR*) was more common in *P. aeruginosa* and *S. maltophilia*. Arsenite efflux (*arsBCR*) was found in a number of species, including *Staphylococcus aureus*, *K. michiganensis*, *K. pneumoniae*, *Escherichia coli*, *Enterobacter hormaechei* and *R. planticola*. Nickel resistance (*ncrABC*) and silver resistance (*SilABEFRS*) were only found in *Klebsiella* sp., with the exception of one sample containing *Pantoea* sp. with *silA*, *silP* and *silR*.

*Pseudomonas* sp. were the main contributors of biocide resistance genes, with *ttgABR* conferring toluene resistance and *sprABRS* involved in gliding motility. Quaternary ammonium compound (qac) resistance genes were identified in a number of species, including *Paenibacillus urinalis*, *Delftia* sp., *Pseudomonas* sp. and *Staphylococcus* sp.

As AMR genes are commonly found on mobile elements, binning is not always effective at linking genes to their presumed host. Here, binning did result in loss of AMR and virulence gene resolution for genes that could not be adequately binned: out of 38 sample sites from T2 that had at least one resistance gene, we found that 22 samples lost AMR or virulence genes through binning, with a median of 5 genes (range 1–30) per sample not reported in binned MAGs.

To estimate the proportion of AMR genes on plasmids vs. the chromosome, we classified MAG contigs into their predicted origin (chromosome, plasmid or virus) using Genomad [25] (Dataset S1). At T1, four AMR genes had chromosomal origin [two *tmexD*, *aac(6′)-I* and *msr(C*)] and one had a predicted plasmid origin (*oqxB*). At T2, 6 out of 267 AMR genes were predicted to be of viral origin. In total, 155 genes (58%) were predicted to be of chromosomal origin and included mostly efflux pumps (65/155, 42%), cephalosporins (14/155, 9%) and beta-lactams (11/155, 7%). In total, 112 genes (42%) were predicted to originate from plasmids, although this number is likely artificially lower than the truth based on the limited ability to bin plasmid contigs. Again, these were mostly genes relating to efflux pumps (27/112, 24%), phenicols (10/112, 9%) and fluoroquinolones (9/112, 8%).

### Cluster analysis from plate sweeps identifies transmission hotspots

To determine if we could identify transmission from hospital plate sweeps, we utilized two methods for clustering of strains from metagenomic data. The first method relies on assembly and binning of MAGs, followed by rapid ANI using approximate mapping with Skani. The second method utilizes a mapping and alignment approach using a large reference database to generate strain-specific multiple sequence alignments across samples and then infer single nucleotide polymorphisms (SNPs) using an empirical Bayes approach (Tracs). Given the lack of similarities between T1 and T2, we focused solely on identifying possible transmission of strains across sites in a single timepoint (T2).

To identify high-confidence clusters using Skani, we required MAGs with >70% completeness (based on CheckM), >99% ANI and an alignment length of at least 70%. Using these thresholds, we identified 13 clusters (≥2 samples) from 29 samples (70%). One cluster was removed, as we could not identify any known bacterial species in the associated MAGs.

Using a threshold of 10 SNPs with Tracs, we identified 17 clusters from 22 samples (54%). There was clear overlap in the clusters detected between both methods, with 70% of clusters from Tracs and 78% of clusters from Skani identifying the same species/sample combinations (Dataset S1). In particular, *Pseudomonas*, *Delftia*, *Paenibacillus* and *Stenotrophomonas* clusters were identified by both methods. Areas of disagreement were the detection of clusters containing *Acinetobacter* (Tracs only), *Staphylococcus* (Skani only) and *Pseudomonas* (both Skani and Tracs).

**Fig. 7.**
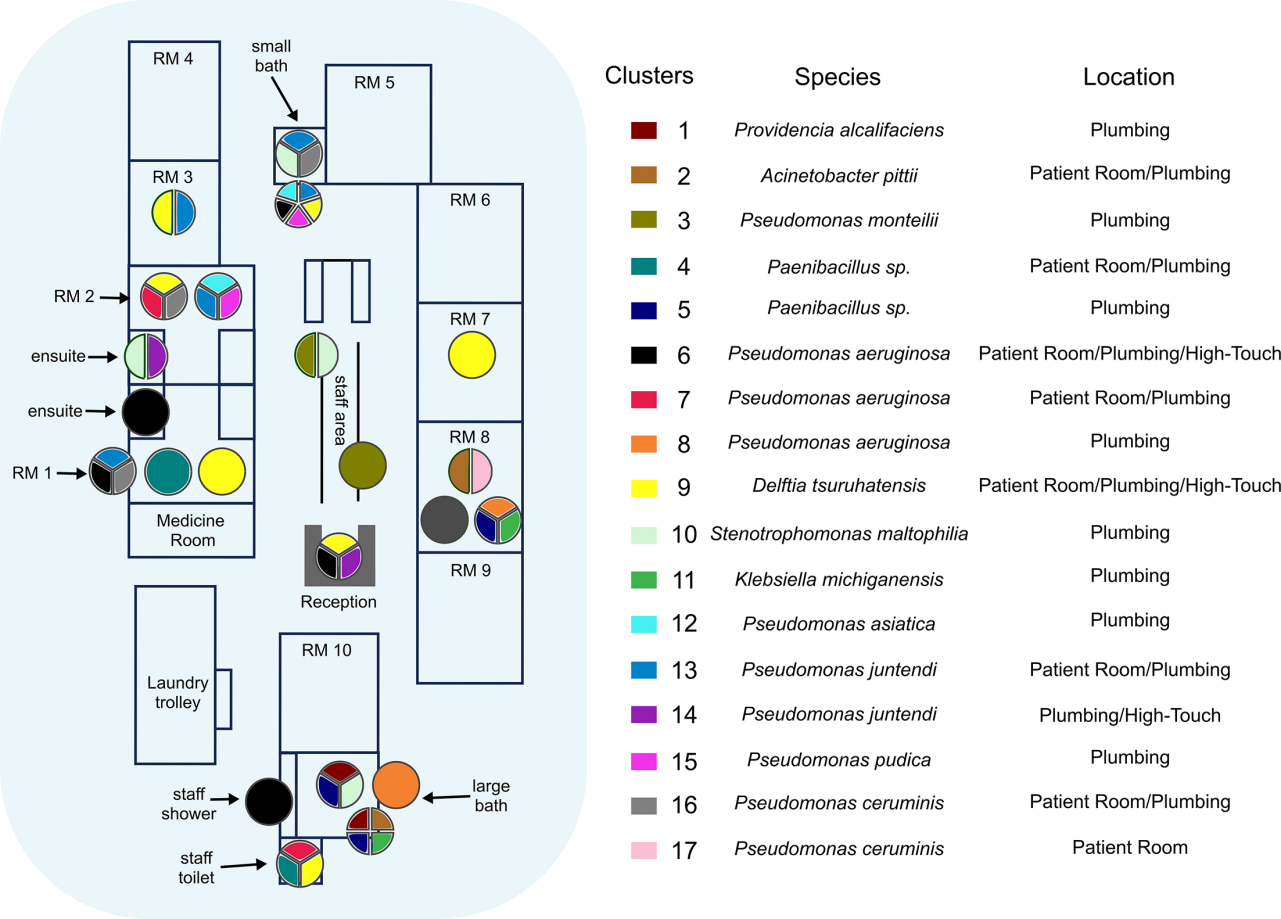
Clustering identified using Tracs: clusters were identified using a SNP threshold of 10. Pie charts represent specific sites within the ICU with multiple transmission clusters detected.

Results from the Skani clustering identified possible contamination from the binning step, as some clustered MAGs had several identified species (*n*=11/51, ~20%, Dataset S1). As such, we moved forward with the interpretation of the Tracs results in the ICU environment (Fig. 7). Hotspots for transmission were identified in several locations, notably both shared bathrooms, the negative pressure rooms (rooms 1 and 2), the staff toilets, the reception desk and room 8. The two sites linked to the most diverse transmission clusters were ICU_S24 (small bathroom toilet rail) and ICU_S60 (large bathroom sink). The most widespread transmission clusters were cluster 9 (*Delftia tsuruhatensis*, 7 sites), followed by cluster 6 (*P. aeruginosa*, 5 sites) and cluster 13 (*Pseudomonas juntendi*, 5 sites).

To identify possible AMR and virulence genes linked to these transmission clusters, we looked at shared AMR genes found across sites with putative transmission (Table 1). Across 17 clusters, we found on average 5 AMR genes (range 0–11) and 12 virulence genes (range 0–31) shared across sites.

**Table 1.** AMR and virulence genes identified across all sampling sites for single clusters

Cluster no.	Sample	AMR gene	Virulence gene
1	ICU_S40, ICU_S60	*blaPLA-2a*, *fosA*, *oqxB*	*ncrY*, *silS*, *arsD*, *silR*, *silP*, *pcoC*, *silF*, *pcoA*, *pcoR*, *ncrC*, *silB*, *pcoD*, *arsA*, *arsB*, *arsC*, *merR*, *merP*, *merD*, *merE*, *merT*, *merA*, *ncrB*, *ncrA*, *fieF*, *silC*, *silE*, *pcoS*, *arsR*, *silA*, *pcoB*
2	ICU_S34, ICU_S60	*amvA*	*merT*, *nreB*, *merD*, *merA*, *merR*, *merE*
3	ICU_S37, ICU_S48	*mexE*	*ttgB*, *ttgR*, *copR*, *cadR*, *chrR*, *ttgA*
4	ICU_S41, ICU_S42		*merR2*, *merT*, *qacH*, *merR1*
5	ICU_S36, ICU_S40, ICU_S60	*oqxB*, *blaPLA-2a*, *fosA*	*silR*, *silB*, *pcoA*, *arsA*, *arsR*, *silE*, *pcoC*, *pcoR*, *pcoS*, *silC*, *merE*, *silS*, *silP*, *silF*, *silA*, *ncrB*, *ncrC*, *ncrY*, *ncrA*, *merR*, *arsC*, *merT*, *pcoB*, *pcoD*, *merP*, *arsB*, *arsD*, *fieF*, *merD*, *merA*
6	ICU_S23, ICU_S24, ICU_S44, ICU_S45, ICU_S52	*aph(3)-IIb*, *fosA*, *blaRSC1*, *blaPDC-8*, *mexX*, *mexE*, *catB7*, *crpP*, *mexA*, *blaOXA-905*	*trxLHR*, *clpK*, *kefB-GI*, *psi-GI*
7	ICU_S41, ICU_S47	*mexA*, *blaPDC-34*, *blaOXA-488*, *fosA*, *catB7*, *mexX*, *aph(3)-IIb*, *crpP*, *mexE*	*cadR*, *merT*, *merE*, *merF*, *merA*, *merR*, *ttgR*, *ttgB*, *ttgA*, *merP*, *merD*
8	ICU_S30, ICU_S36	*mexA*, *blaPDC-108*, *blaOXA-50*, *aph(3)-IIb*, *mexX*, *catB7*, *fosA*, *mexE*	
9	ICU_S24, ICU_S33, ICU_S41, ICU_S47, ICU_S52, ICU_S54, ICU_S61		*merE*, *merD*, *merR*, *merT*, *merP*, *merA*
10	ICU_S25, ICU_S40, ICU_S43, ICU_S48	*emrB*, *aph(3)-IIc*, *emrC*, *mexE*, *smeF*, *blaL1*, *emrA*	*ttgB*, *copB*, *copL*, *ttgR*, *copA*
11	ICU_S36, ICU_S60	*aac(6)*, *emrD*, *aph(3)-Ia*, *blaOXY*, *blaPLA-2a*, *oqxA*, *blaSRT*, *fosA*, *oqxB*	*pcoB*, *merT*, *arsC*, *silA*, *ncrB*, *merD*, *silR*, *pcoS*, *silF*, *fieF*, *ncrY*, *merA*, *merR*, *silE*, *pcoA*, *merP*, *arsB*, *arsR*, *silB*, *ncrC*, *pcoR*, *pcoD*, *silS*, *ssmE*, *silP*, *ncrA*, *silC*, *arsA*, *arsD*, *pcoC*, *merE*
12	ICU_S24, ICU_S46	*aph(3)-IIb*, *sul1*, *tet(C)*, *fosA*, *mexA*, *ant(2)-Ia*, *crpP*, *aadA2*, *aadA6*, *catB7*, *mexE*	*merA*, *ttgR*, *ttgB*, *srpA*, *merE*, *ttgA*, *qacEdelta1*, *merD*, *ttgI*, *cadR*, *merT*, *merP*, *srpR*, *merF*, *srpB*, *merR*, *srpS*
13	ICU_S24, ICU_S43, ICU_S45, ICU_S46, ICU_S54	*mexE*	*ttgA*, *srpS*, *merE*, *ttgR*, *merR*, *cadR*, *ttgI*, *srpR*, *ttgB*, *srpB*, *merD*, *srpA*
14	ICU_S25, ICU_S52	*emrB*, *aph(6)*, *sul1*, *aadA2*, *trxLHR*, *aph(3)-Ia*, *emrA*, *aac(3)-Id*, *mexE*	*ttgR*, *qacEdelta1*, *srpB*, *ttgI*, *psi-GI*, *copB*, *ttgA*, *srpA*, *clpK*, *srpS*, *cadR*, *ttgB*, *srpR*, *kefB-GI*
15	ICU_S24, ICU_S46	*catB7*, *sul1*, *aph(3)-IIb*, *tet(C)*, *crpP*, *ant(2)-Ia*, *aadA6*, *mexA*, *mexE*, *aadA2*, *fosA*	*merT*, *ttgA*, *merF*, *srpB*, *ttgR*, *merR*, *merP*, *merD*, *merA*, *srpR*, *ttgI*, *srpA*, *ttgB*, *merE*, *cadR*, *qacEdelta1*, *srpS*
16	ICU_S43, ICU_S45, ICU_S47	*mexE*	*ttgB*, *merE*, *merR*, *merD*, *ttgR*, *cadR*, *ttgA*
17	ICU_S34, ICU_S59	*mexE*	*ttgB*, *ttgI*, *srpR*, *srpB*, *srpS*, *ttgA*, *cadR*, *ttgR*, *srpA*

## Discussion

Here, we show the rapid change in the environmental microbiome of a newly established ICU pre- and post-patient introduction using a non-selective plate sweep approach. We show that this approach is sensitive enough to detect AMR and virulence genes as well as possible transmission events and could be a powerful tool for proactive surveillance of the hospital environment.

When it comes to understanding the full genetic diversity of a bacterial sample, the limitation of single colony picks is well established. Even for presumed monocultures of a single infectious organism, deep sequencing of plate sweeps has revealed a plethora of genetic variability [29]. Characterizing within-species genetic variation has been computationally feasible for more than a decade [30] and has been used across multiple studies to reveal a more accurate representation of the intra-patient bacterial community. However, in most cases, these approaches have focused solely on understanding genetic variation of single bacterial species. Indeed, tools such as mSweeps [31] and to some extent mGEMS [32] lean towards a narrow focus on particular species, usually accomplished via growth on selective media [11,14, 33], although they can be utilized for mixed species samples with an appropriate reference database. Non-selective plate sweep approaches in non-patient settings have been performed previously [15] and often coined ‘quasi-metagenomics’, particularly in the foodborne pathogen surveillance field [34,35]. Apart from *Mycobacterium tuberculosis*, the leading bacterial species associated with either human pathogenesis and/or environmental transmission in hospitals are readily culturable in laboratory settings, including many of the WHO priority pathogens. As such, employing direct metagenomics over enrichment methods is likely only revealing a small fraction of additional pathogenic potential in the hospital environment. Furthermore, intermediate culture enrichment significantly reduces the cost and manual handling involved compared to standard direct-from-sample metagenomics [36], making it a more feasible ongoing method of surveillance. While not completed here, enrichment can also lead to more detailed assessment of the microbial community, as the lower complexity/higher coverage samples are more accessible to long-read sequencing technologies such as Oxford Nanopore, which has no native amplification step, and could be used to generate complete length reference genomes and mobile elements (such as phage and plasmids) [15].

In this study of the ICU pre- and post-patient introduction, we highlight two main findings: (1) microbes were present in the environment pre- and post-patient introduction, but the post-patient environment replaced the initial microbial communities, bringing with it substantial AMR capabilities and (2) regardless of timepoint, virulence capacity, particularly metal resistance, was essential. To date, several studies have observed the change in environmental microbiomes in new or refurbished healthcare settings [37,38], with a primary focus on methicillin-resistant *Sta. aureus* [39,40] and Enterococci [41]. Here, we show that a diverse range of bacteria can be tracked in the hospital environment without selective pressure. While not applied here, an advantage of our method is that it is possible to observe acquisition of resistance genes in pre-existing sensitive environmental microbes, thus noticing evolution of AMR in the hospital environment. Additionally, we show that identifying transmission hotspots using non-resistant microbes can also provide critical information to infection control staff prior to an outbreak of a more serious pathogenic bacterium.

With the recent development and release of Tracs [27], we show its utility in providing clear and actionable transmission links from hospital environment plate sweep data. We found that binning with Metabat2 was insufficiently sensitive to provide clear transmission information, despite sharing similarities in clustering to Tracs. We considered the use of Semibin2 [42]; however, the requirement for a trained machine learning model was somewhat impractical for our purposes, as we did not have prior knowledge of all species expected to be encountered in the hospital environment. An additional technical limitation we discovered was that while binning improved estimation of origin for some resistance genes, many resistance genes were also lost due to the inability to confidently place them (and other mobile genetic elements) into appropriate species bins.

Unsurprisingly, we found that plumbing was a major source of pathogenic bacteria and AMR/virulence genes. This has been reported numerous times across various healthcare settings [43,44]. We also found a number of key species routinely detected in hospital environments, including *P. aeruginosa* [45] and *S. maltophilia* [46,47]. The rapid acquisition of these opportunistic pathogens, which contain intrinsic resistance to a number of antimicrobials, may assist the survival of other species in the hospital environment. *Pseudomonas* sp. in particular was found in conjunction with *Acinetobacter*, *Stenotrophomonas*, *Klebsiella*, *Raoultella*, *Serratia* and *Brevundimonas* and has been implicated in co-infections with other microbes due to its proclivity for biofilm formation [48].

There were several limitations of this study. Firstly, we were unable to collect samples from patients or continue environmental surveillance beyond our two timepoints. As such, we do not know what the stability of this environmental microbiome will be over time or how tightly linked it is to the patient cohort. Secondly, we could not perform isolate or metagenomic sequencing alongside to determine the robustness of the plate sweep approach in providing a suitable approximation of the pathogenic potential within the environment. We were also unable to sequence all available samples at timepoint 1 due to logistical problems; however, this only impacted 5/78 sites (RM6 handle, RM8 equipment, staff toilet sink, staff sink 2 and LRG bathroom sink). We also acknowledge that competition and overgrowth on the plates will disrupt the actual proportion of bacteria and are therefore not representative of the real population ratios. We did not explore anaerobic growth as a condition, but acknowledged that this could be an easily incorporable element to this type of surveillance. Finally, slow-growing or fastidious organisms of relevance in the environment (e.g. non-tuberculous mycobacteria) will not be captured using this technique.

In summary, we present a pilot study demonstrating hospital environmental surveillance using plate sweeps in a newly built ICU in regional Queensland, Australia. We identified rapid colonization of the ICU environment post-patient introduction with multiple pathogenic bacteria. Non-selective enrichment enabled tracking of bacteria around the ICU, identifying key hotspots for transmission linked mainly to bathrooms. Implementation of this surveillance is cost-effective enough to be performed routinely and can identify target areas for additional infection control measures before an outbreak takes place.

## Supplementary material

10.1099/mgen.0.001597 Uncited Supplementary Material 1.

10.1099/mgen.0.001597 Uncited Supplementary Material 2.

## Abbreviations

AMR: antimicrobial resistance
ANI: average nucleotide identity
GTDB: genome taxonomy database
HAIs: healthcare-associated infections
HBA: Horse Blood agar
ICU: intensive care unit
LB: Luria–Bertani
MAC: MacConkey agar
MAGs: metagenome-assembled genomes
NA: nutrient agar
WGS: whole genome sequencing
